# Infiltrating CD4+ T cells attenuate chemotherapy sensitivity in prostate cancer via CCL5 signaling

**DOI:** 10.1002/pros.23810

**Published:** 2019-04-24

**Authors:** Peng Xiang, Song Jin, Yang Yang, Jindong Sheng, Qun He, Yi Song, Wei Yu, Shuai Hu, Jie Jin

**Affiliations:** ^1^ Department of Urology, Peking University First Hospital and Institute of Urology Peking University Beijing China; ^2^ National Research Center for Genitourinary Oncology Beijing China; ^3^ Beijing Key Laboratory of Urogenital Diseases (Male) Molecular Diagnosis and Treatment Center Beijing China

**Keywords:** C‐C motif chemokine ligand 5 signaling, CD4+ T cells, chemotherapy resistance, PCa

## Abstract

**Background:**

Chemotherapy with Docetaxel (Doc) is efficient in a subset of prostate cancer (PCa) cases; however, most patients ultimately develop resistance to Docetaxel. The tumor immune microenvironment and secreted cytokines play a substantial role in development of resistance to chemotherapy. Our previous study has demonstrated that CD4+ T cells in prostate tumor microenvironment contribute to PCa progression; meanwhile, we found increased CD4+ T‐cell infiltration in tumor area after Doc treatment; however, their effects on PCa chemosensitivity remain unclear. Here, we aim to explore the role and mechanisms of CD4+ T cells in PCa chemotherapy sensitivity.

**Methods:**

CD4+ T‐cell infiltration in Doc‐treated paraffin‐embedded specimens from transurethral resection of prostate, radical prostatectomy, or bone metastasis was detected by immunohistochemistry. The castration‐resistant PCa cell lines—C4‐2 and CWR22RV1, and CD4+ T‐cell lines—HH and Molt‐3 were used in the coculture system. After coculture with the lymphocytes, PCa cell chemosensitivity was detected by cell counting kit‐8, terminal deoxynucleotidyl transferase dUTP nick‐end labeling assays, and Western blot analysis. Various cell cytokines were determined by cytokine arrays and reverse‐transcription polymerase chain reaction. The recombinant human C‐C motif chemokine ligand 5 (CCL5) was added to PCa cells for further confirming its effects and anti‐CCL5 antibody was used for neutralization. S3I‐201, a signal transducer and activator of transcription 3 (STAT3) inhibitor, was added to the coculture system to detect STAT3 role in chemosensitivity. Tumor xenografts in nude mice were used for confirming effects of CD4+ T cells in vivo study.

**Results:**

We found more infiltrated CD4+ T cells in human PCa lesions than in the adjacent noncancerous tissues after Doc treatment. In vitro cell line study confirmed that CD4+ T cells increase the PCa Doc resistance. Quantative polymerase chain reaction and cytokine arrays indicated that after coculture with PCa, CD4+ T cells could secrete large amounts of CCL5. Moreover, CCL5 stimulation enhanced PCa resistance to Doc, and anti‐CCL5 antibody could partly reverse this process. We found that CD4+ T cells could activate P‐STAT3 signaling via secreting CCL5 and adding a STAT3 inhibitor can reverse the chemoresistance. In vivo mouse model with xenografted 22RV1 cells and CD4+ T cells also confirmed the in vitro results.

**Conclusions:**

Together, our results indicate that infiltrating CD4+ T cells could promote PCa chemotherapy resistance via modulation of the CCL5/STAT3 signaling pathway.

**Abbreviations**
AR: androgen receptor
CCL5: C‐C motif chemokine ligand 5
CRPC: castration‐resistant prostate cancer
Doc: Docetaxel
PCa: prostate cancer

## 1 INTRODUCTION

Docetaxel (Doc) is currently one of the standard first‐line therapies for patients with castration‐resistant prostate cancer (CRPC).^1^, ^2^ While CRPC is generally a Doc‐sensitive disease, there is a large variability in its response because of inherent or acquired Doc resistance. Approximately half of all patients do not respond to Doc and those who do eventually develop resistance to Doc within 24 months of initial exposure.^3^, ^4^

Resistance to Doc is poorly understood and may be caused by a number of mechanisms. These mechanisms may include androgen receptor (AR) signaling, activation of prosurvival pathways, and the acquisition of a cancer stem cell morphology.^5^, ^6^, ^7^, ^8^ Further, tumor immune microenvironment and overexpression of inflammation‐associated molecules have an important role in the development of Doc resistance.^7^, ^8^

Among infiltrating immune cells, innate and adaptive immune cells were shown to significantly correlate with PCa aggressiveness.^9^, ^10^, ^11^ Moreover, mast cells could enhance PCa resistance to chemotherapy and radiotherapy via activation of p38/p53/p21 and ATM protein kinase signals.^12^ Similarly, cytokines from immune cells also affect chemotherapy resistance, such as interleukin 6 (IL6), IL8, CCL2, and transforming growth factor‐β1.^8^, ^13^

T cells, especially CD4+ T cells, are an important part of the tumor immune inflammatory microenvironment. Accumulating evidence suggests that CD4+ T cells could contribute to a tumor immune evasion and tumor progression.^14^, ^15^ Our previous study has shown that CD4+ T cells in the prostate tumor microenvironment contribute to PCa progression,^10^ and we found increased CD4+ T‐cell infiltration in PCa tissue after Doc treatment. However, their effects on PCa chemosensitivity remain unclear. Here, we studied the role of infiltrating CD4+ T cells in PCa chemotherapy sensitivity.

## 2 MATERIALS AND METHODS

### 2.1 Patients

We recruited 15 patients whose prostate biopsies showed clinical evidence of PCa, and who received Doc treatment. These paraffin‐embedded specimens from radical prostatectomy, transurethral resection of prostate (TURP), or bone metastasis. Patients with CRPC received Doc treatment often show local progression and then suffer from urinary obstruction due to tumor growth. In these patients, transurethral resection of the tumor often helps them to regain normal voiding function. In our study, TURP specimens were also selected. Pathologically confirmed prostate carcinoma bone metastasis specimens also were obtained from patients that had undergone Doc treatment. These patients fulfilled CRPC criteria according to the 2018 European Association of Urology guideline for PCa (castration levels of serum testosterone which might present as one or any combination of continuous rising prostate‐specific antigen [PSA], new metastatic lesions, or new clinical symptoms).^16^ Each patient biopsy tissue was divided into two parts: PCa area and adjacent noncancerous area. The diagnosis of PCa area and adjacent noncancerous area were confirmed by two pathologists. All clinical data analyses and prostate specimen collection were performed after obtaining informed consent from all patients and approval of the Peking University First Hospital Institutional Review Board. Patient information is included in Table 1.

**Table 1:** The clinical information from PCa patients

No.	Age, y	Gleason score	PSA before Doc, ng/mL	PSA after Doc, ng/mL	Metastasis before treatment	Metastasis after treatment	Docetaxel dose	Frequency
1	64	5 + 4	0.33	5.62	Bladder and seminal vesicle metastasis	Multiple metastases and bone pain	120 mg	6
2	82	5 + 4	140	170.4	bone metastases	Multiple bone metastases and fracture	100 mg	5
3	56	5 + 4	25.99	51.94	T4 metastases	Multiple bone metastases and spinal compression	120 mg	16
4	56	5 + 4	445	795.8	Multiple bone metastases	Multiple metastases, paraplegia and death	120 mg	4
5	65	5 + 4	134.2	301.4	Multiple bone metastases	Multiple metastases and spinal compression	120 mg	7
6	68	5 + 4	28.11	35.35	Multiple bone metastases	Multiple metastases and spinal compression	120 mg	2
7	61	4 + 5	30.43	80.12	T12 metastases	Multiple metastases and bone pain	120 mg	17
8	62	4 + 5	25	1393	Right sciatic metastasis	Multiple metastases and pathological fracture	140 mg	11
9	62	4 + 5	32.65	371.9	T8‐T10 metastases	Multiple metastases and bone pain	140 mg	6
10	63	5 + 4	126.56	523.7	Multiple bone metastases	Multiple bone metastases and bone pain	140 mg	20
11	55	5 + 4	48.1	180.6	None	Multiple bone metastases and bone pain	120 mg	10
12	63	4 + 5	28.11	16.25	T12 and L2 metastasis	Multiple bone metastases and bone pain	120 mg	3
13	66	4 + 4	481.1	23.54	Bone metastasis	Multiple bone metastases and bone pain	140 mg	16
14	71	5 + 5	504.9	25.43	None	Multiple bone metastases and bone pain	120 mg	2
15	49	4 + 3	26.78	0.006	L3 metastasis	Multiple bone metastases and fracture	120 mg	5

Abbreviations: Doc, Docetaxel; PCa, prostate cancer; PSA, prostate‐specific antigen.

### 2.2 Cell culture

C4‐2 and CWR22Rv1 cell lines were purchased from the American Type Culture Collection (Rockville, MD) and grown in Rosewell Park Memorial Institute (RPMI)‐1640 medium containing 1% penicillin and streptomycin, supplemented with 10% fetal bovine serum (FBS). CD4+ T‐lymphocytic cell lines HH and Molt‐3 were acquired from the American Type Culture Collection (Rockville, MD) and maintained in 10% heat‐inactivated FBS, RPMI media with 1% pen/strep. All cell lines were cultured in a 5% CO_2_ humidified incubator at 37℃.

### 2.3 Reagents and materials

Recombinant human C‐C motif chemokine ligand 5 (CCL5) (RANTES) was purchased from PeproTech, Inc, (300‐06; Rocky Hill, NJ), and anti‐CCL5 neutralizing antibody was purchased from Abcam (ab9679; Cambridge, UK). Doc was from Selleck Chemicals (S1148; Houston, TX). S3I‐201 was obtained from Selleck Chemicals (S1155). For CCL5, anti‐CCL5, and S3I‐201 treatment, stocks were adjusted to a final concentration of 100 ng/mL, 1 μg/mL, and 10 μM, respectively.

### 2.4 Cell proliferation assay

C4‐2/CWR22RV1 cells at 1 × 10^4^ cells/well were plated into the lower chamber of the 24‐well transwell plates overnight. Subsequently, HH/Molt‐3 cells at 1 × 10^4^ cells/well were plated onto the upper chamber with 0.4 µm pore polycarbonate membrane inserts in the coculture group, or only had fresh media added into the upper chamber in the monoculture group. During a 48‐hour coculture, they were treated with indicated doses of drugs in normal media for 24 or 48 hours. C4‐2/CWR22RV1 cell growth status was detected by the Cell Counting Kit‐8 (CCK8) according to the manufacturer's protocol. The absorbance value was measured using a Varioskan Flash (Thermo Fisher Scientific, Waltham, MA) at 450 nm.

### 2.5 Fluorescent in situ detection of DNA fragmentation (Tunel)

Apoptotic cell death was determined using terminal deoxynucleotidyl transferase dUTP nick end labeling (Tunel) staining with an In Situ Cell Death Detection Kit (Roche Molecular Biochemicals, Indianapolis, IN) following the manufacturer's protocol. Tunel‐positive cells were calculated as the number of positive cells divided by the total number of cells/field in 10 random fields at ×200 magnification.

### 2.6 Quantitative polymerase chain reaction

Total RNA was extracted from each cell line using TRIzol (15596‐018; Invitrogen, Grand Island, NY). Reverse transcription was performed using the iScript reverse transcription kit (Bio‐Rad, Hercules, CA). Quantitative real‐time polymerase chain reaction (qRT‐PCR) was conducted using a Bio‐Rad CFX96 system with SYBR green to determine the level of messenger RNA expression of a gene of interest. Expression levels were normalized to the expression of glyceraldehyde 3‐phosphate dehydrogenase (GAPDH) RNA. The following primer sequences were used: CCL5, F: CCAGCAGTCGTCTTTGTCAC, R: CTCTGGGTTGGCACACACTT; and GAPDH, F: TGTGGGC ATCAATGGATTTGG, R: ACACCATGTATTCCGGGTCAAT.

### 2.7 Western blot analysis

The expressions of specific genes were determined by Western blot analysis according to a previous study.^10^ Briefly, cells were lysed in radioimmunoprecipitation assay buffer, and proteins (20 µg) were separated on 8 to 10% sodium dodecyl sulfate‐polyacrylamide gel electrophoresis gel and then transferred onto polyvinylidene fluoride membranes (P90719, Millipore, MA). After blocking membranes, they were incubated with appropriate dilutions (1:1000) of specific primary antibodies, the blots were then incubated with horseradish peroxidase‐conjugated secondary antibodies and visualized using the ECL system (Thermo Fisher Scientific, Waltham, MA). The following primary antibodies were used: rabbit anti‐cleaved poly ADP ribose polymerase (PARP) (CST 9541), Signal transducer and activator of transcription 3 (STAT3) (CST 12640), and P‐STAT3 (Tyr705) (CST 9145) from Cell Signaling Technology (Danvers, MA) and anti‐GAPDH from Santa Cruz Biotechnology (Santa Cruz, CA).

### 2.8 Cytokine arrays

(Conditioned mediums) CMs were collected from C4‐2 culture only, HH culture only, and C4‐2‐HH coculture for 48 hours. Briefly, 1 mL of these supernatants was incubated with the RayBio1 Human Cytokine Antibody Array 3 (Catalog no. AAH‐CYT‐3) (RayBiotech, Norcross, GA). Arrays were processed according to the manufacturer's protocols and evaluated using the chemiluminescence system (Thermo Fisher Scientific).

### 2.9 Immunohistochemistry

The prostate tumor samples from patients and mice were fixed in 4% neutral buffered paraformaldehyde and embedded in paraffin. The primary antibodies of anti‐Ki67 (ab15580; Abcam), anti‐cleaved PARP (CST 9541), anti‐CCL5 (ab9679; Abcam), and anti‐P‐STAT3 (CST 9145), were used for staining. The primary antibody was recognized by the biotinylated secondary antibody (Vector, Burlingame, CA) and visualized by the VECTASTAIN ABC peroxidase system and peroxidase substrate 3,3′‐diaminobenzidine kit (Vector).

### 2.10 Isolation of CD4+ T cells and flow cytometry

Human peripheral blood mononuclear cells (PBMCs) were isolated from the whole blood of healthy patients, using Ficoll‐Paque (Biochrom, Berlin, Germany) density gradient centrifugation. Then PBMCs incubated with CD4‐APC antibody (BD Biosciences, CA) for 20 minutes. Human CD4+ T cells were isolated from PBMCs via BD influx flow cytometry (BD Biosciences). CD4+ T cells were cultured in RPMI‐1640 containing 10% FBS supplemented with antibiotics.

### 2.11 In vivo subcutaneous implantation and Doc administration studies

Male nude mice (6‐ to 8‐week‐old) were used to create the animal model. In brief, 2 × 10^6^ CWR22Rv1 cells with 2 × 10^5^ HH cells mixed with Matrigel were injected subcutaneously in the right axillary region of 5 mice (CWR22Rv1/HH). Further, 2 × 10^6^ CWR22Rv1 cells mixed with Matrigel (356234, BD, NJ) were injected subcutaneously in the right axillary region of another five mice (CWR22Rv1). After xenograft tumors grew to about 50 mm^3^, five of the CWR22Rv1/HH mice and five of the CWR22Rv1 mice received intraperitoneal Doc administration at 30 mg/kg once per week for 3 weeks. The tumor volume was calculated as follows: length × width^2^ × 1/2. Following, the mice were killed and subcutaneous tumors were harvested for immunohistochemistry (IHC) studies. All animal studies were performed under the supervision and guidelines of the Peking University First Hospital Animal Care and Use Committee. A similar method for C4‐2 response in the vivo study.

### 2.12 Statistics

All statistical analyses were carried out with SPSS 19.0 (SPSS Inc, Chicago, IL). The data values were presented as the mean ± standard deviation. Differences in mean values between each group were analyzed by the two‐tailed Student test, and the mean values of more than two groups were compared by one‐way analysis of variance. *P* < 0.05 was considered statistically significant. All experiments were repeated three times.

## 3 RESULTS

### 3.1 CD4+ T‐cell infiltration in PCa patients with Doc treatment and altered PCa chemotherapy sensitivity

**Figure 1:**
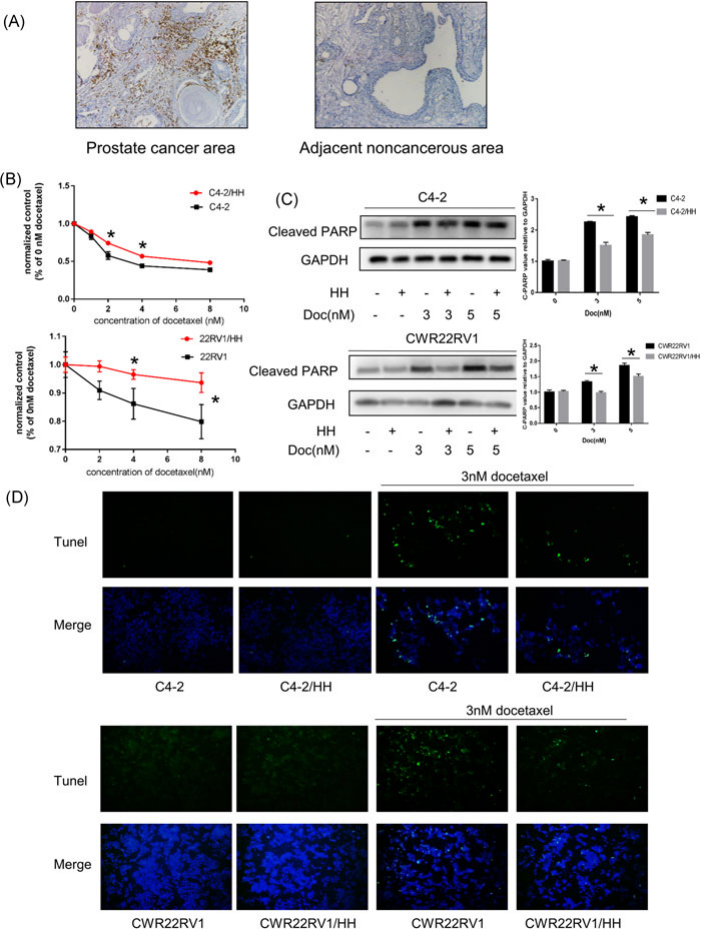
CD4+ T**‐**cell infiltration in PCa patients with Doc treatment and altered PCa chemotherapy sensitivity. A, Clinical specimen IHC staining from Doc treatment PCa patients showed that more CD4+ T cells infiltrated the prostate cancer area than in the adjacent noncancerous area. B, PCa cells were cocultured with HH cells for 2 days in 24‐well plates under 0.4 μm transwell membranes; a total of 1 × 10^5^ PCa, C4‐2/CWR22Rv1, were plated in lower chambers of transwells and 1 × 10^5^ CD4+ T cells, HH were plated on the upper insert chambers. Subsequently, cells were treated with different doses of Doc for 48 hours and tested with CCK8. Consider absorbance of 0 nM as control, all absorbance of other doses was compared to control. After coculture with HH cells, C4‐2 cells become more resistant to Doc treatment. Data are presented as mean ± SD, *n* = 3. *P < 0.05 vs control. C, Western blot analysis: for Doc treatment, PCa cells cocultured with HH cells showed less expression of cleaved PARP compared to PCa cells without coculture HH cells. Data are also presented as mean ± SD, *n* = 3. *P < 0.05 vs control. D, Tunel assay analysis of cell apoptosis. For Doc treatment, PCa cells cocultured with HH cells showed less cell apoptosis compared to PCa cells without coculture HH cells. CCK8, cell counting kit‐8; Doc, Docetaxel; GAPDH, glyceraldehyde 3‐phosphate dehydrogenase; IHC, immunohistochemistry; PARP, poly ADP ribose polymerase; PCa, prostate cancer; Tunel, terminal deoxynucleotidyl transferase dUTP nick‐end labeling [Color figure can be viewed at wileyonlinelibrary.com]

To examine the potential impact of CD4+ T cells on chemosensitivity on the neighboring PCa, we first compared CD4+ T‐cell infiltration in PCa clinical specimens after chemotherapy using IHC staining. The results showed increased CD4+ T‐cell infiltration in the tumor area than in adjacent noncancerous tissues in Doc‐treated paraffin‐embedded specimens from PCa patients (Figure 1A).

**Figure 2:**
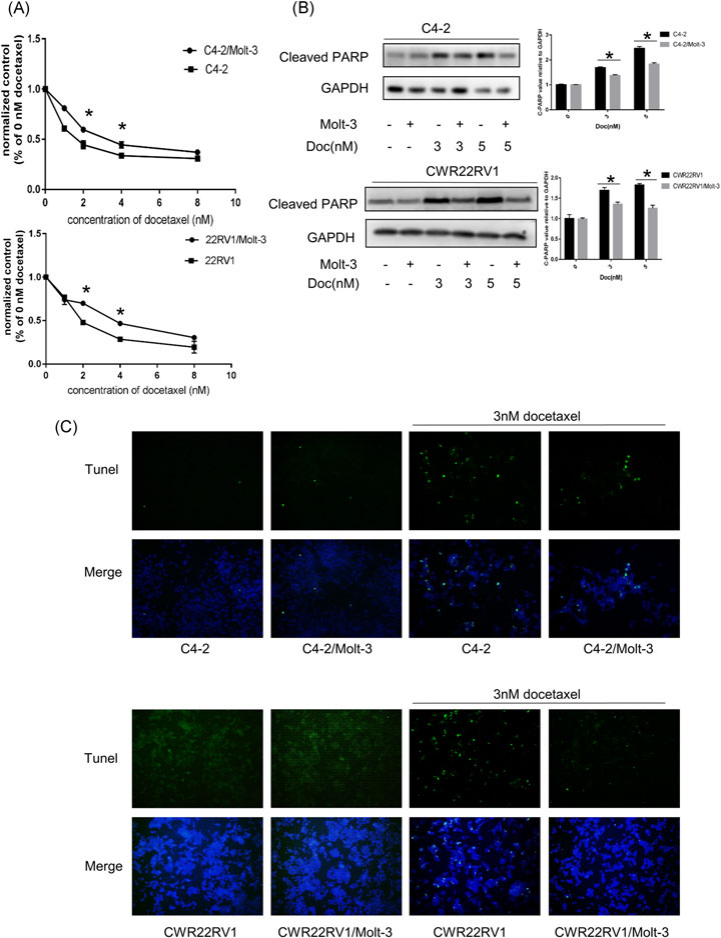
CD4+ T‐cell infiltration in PCa patients with Doc treatment and altered PCa chemotherapy sensitivity. A‐C, Similar results were also obtained when we replaced the HH cells with another CD4+ T‐cell line (Molt‐3 cells). Data are presented as mean ± SD, *n* = 3. *P < 0.05 vs control. Doc, Docetaxel; GAPDH, glyceraldehyde 3‐phosphate dehydrogenase; PARP, poly ADP ribose polymerase; PCa, prostate cancer [Color figure can be viewed at wileyonlinelibrary.com]

To further study the effect of CD4+ T cells on the chemosensitivity of neighboring PCa cells, we applied the coculture system. Results showed that after coculture with CD4+ T cells (HH cells), C4‐2 and 22Rv1 cells became more resistant to Doc treatment in a dose‐dependent manner ranging from 1 nM to 8 nM by CCK8 (Figure 1B). The level of cleaved PARP, another indicator of apoptosis, was also decreased when coculture with HH cells (Figure 1C). Furthermore, we indicated that CD4+ T cells inhibit Doc‐induced apoptosis in C4‐2 and CWR22RV1 cells through the Tunel assay (Figure 1D). Similar results were also obtained when HH cells were replaced with another CD4+ T cell—Molt‐3 cells (Figure 2A‐C). Using CD4+ T cells, which from peripheral blood mononuclear cells also increase PCa cell chemotherapy resistance (Figure S1).

Together, these results suggest that infiltrating CD4+ T cells could decrease PCa chemotherapy sensitivity to Doc.

### 3.2 CCL5 is secreted from CD4+ T cells after coculture with PCa and plays a pivotal role in inducing PCa chemoresistance

**Figure 3:**
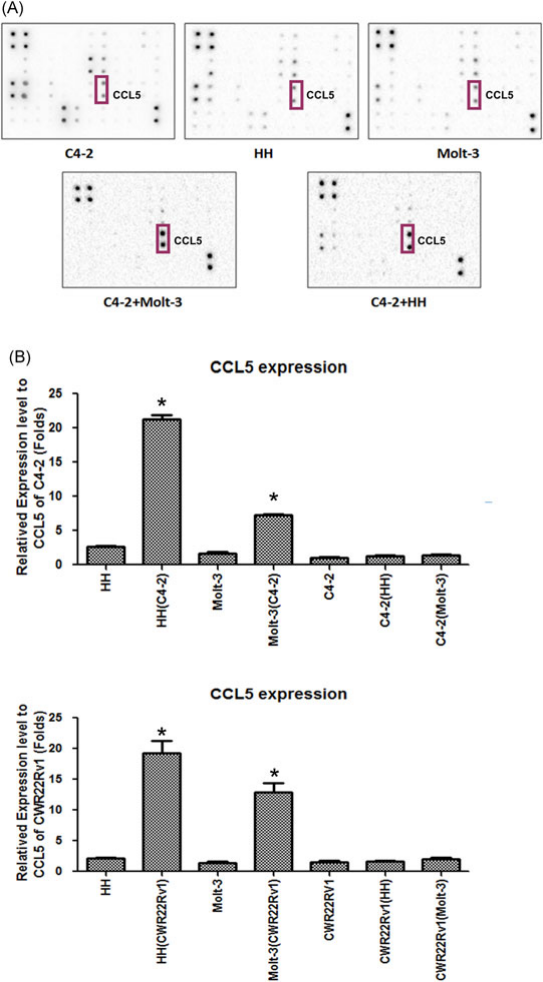
CCL5 is highly secreted from CD4+ T cells after coculture with PCa and plays a pivotal role in inducing PCa chemoresistance. A, Cytokine chip was used to screen high expression and specific cytokines; CCL5 was increased in cocultured conditioned medium compared to control medium. B, RT‐PCR analysis was used to detect related cytokines, and the results showed that CCL5 was highly expressed in cocultured CD4+ T cells. Data are presented as mean ± SD, *n* = 3. *P < 0.05 vs control. CCL5, C‐C motif chemokine ligand 5; GAPDH, glyceraldehyde 3‐phosphate dehydrogenase; PCa, prostate cancer; RT‐PCR, real‐time polymerase chain reaction [Color figure can be viewed at wileyonlinelibrary.com]

To determine which factors influence acquired Doc resistance after coculture with PCa, we compared the expression of some cytokines related to chemoresistant cancer. Cytokine/chemokine assay was performed and found that CCL5 was significantly increased after coculture with CD4+ T cells compared with the control medium (Figure 3A). Further, we found that CCL5 expression was increased in CD4+ T cells after coculture with PCa by qPCR (Figure 3B).

**Figure 4:**
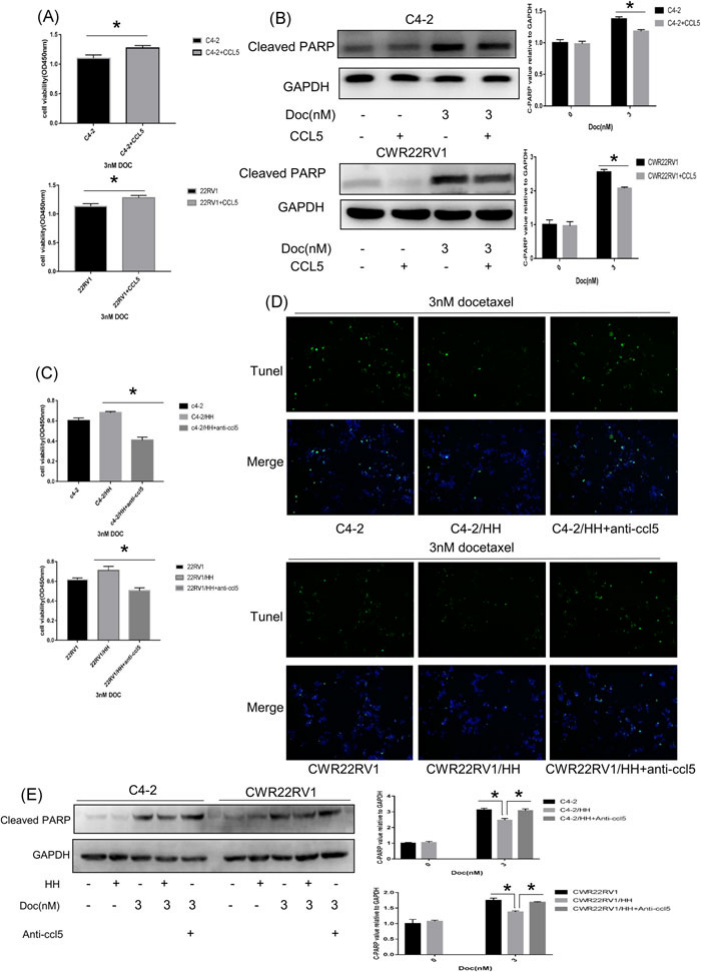
CCL5 is highly secreted from CD4+ T cells after coculture with PCa and plays a pivotal role in inducing PCa chemoresistance. A, B, C4‐2 and 22RV1 cells were pretreated with recombination human CCL5 (100 ng/mL) 24 hours and then treated with 3 nM Doc for 48 hours. Cell viability was detected using CCK8, Data are presented as mean ± SD, *n* = 3. *P < 0.05 vs control. Western blot analysis was used to detect apoptosis‐related proteins levels. Data are also presented as mean ± SD, *n* = 3. *P < 0.05 vs control. CCL5 stimulation enhances PCa Doc resistance. C‐E, C4‐2 and 22RV1 cells were pretreated with anti‐CCL5 (1 μg/mL) and HH/Molt‐3 cells 24 hours and then treated with 3 nM Doc for 48 hours. Cell viability was detected using CCK8, Data are presented as mean ± SD, *n* = 3. *P < 0.05 vs control. Tunel assay analysis was used to detected cell apoptosis. Western blot analysis was used to detect apoptosis‐related proteins levels. Data are also presented as mean ± SD, *n* = 3. *P < 0.05 vs control. Anti‐CCL5 could reverse the effects of CD4+ T cells on PCa chemoresistance. CCK8, cell counting kit‐8; CCL5, C‐C motif chemokine ligand 5; Doc, Docetaxel; GAPDH, glyceraldehyde 3‐phosphate dehydrogenase; PCa, prostate cancer; Tunel, terminal deoxynucleotidyl transferase dUTP nick‐end labeling [Color figure can be viewed at wileyonlinelibrary.com]

To verify whether CCL5 is one of the key factors released from CD4+ T cells to increase neighboring PCa chemoresistance, we directly added CCL5 recombinant proteins into C4‐2 and CWR22RV1 cells. C4‐2 and CWR22RV1 cells were more resistant to Doc after CCL5 treatment compared with the control groups (Figure 4A). Furthermore, the level of cleaved PARP also decreased (Figure 4B). Moreover, a neutralizing antibody against CCL5 was added into the coculture system. The CCK8 assay, Tunel, and Western blot analysis indicated that neutralizing against CCL5 antibody could reverse the effects of CD4+ T cells on PCa chemoresistance (Figure 4C‐E). These results support that CCL5 stimulation enhances Doc resistance.

### 3.3 CD4+ T cells induce PCa chemoresistance via CCL5/P‐STAT3 signals

**Figure 5:**
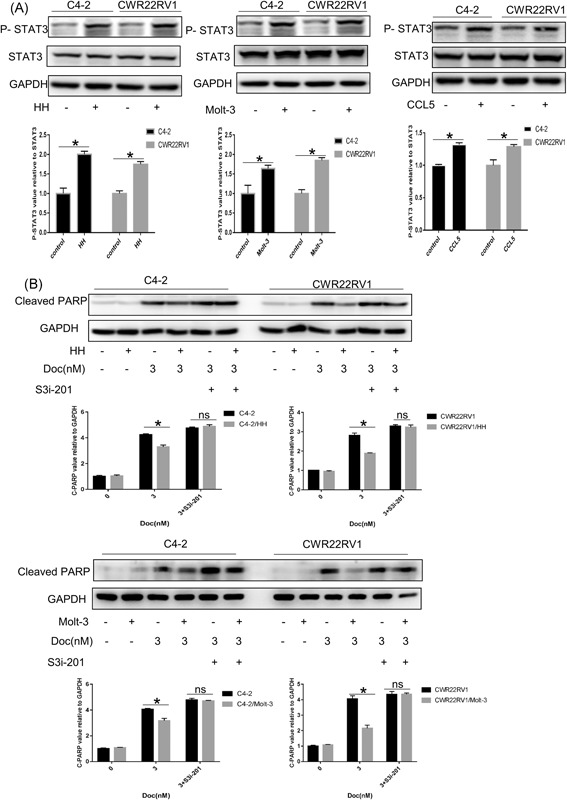
CD4+ T cells induced chemoresistance of PCa via CCL5/P‐STAT3 signaling. A, C4‐2 and 22RV1 cells were treated with CCL5 for 48 hours. Western blot analysis shows that phosphorylation of STAT3 (Tyr705) is increased. Similarly, after coculture with HH cell for 48 hours, phosphorylation of STAT3 (Tyr705) was also increased. Data are also presented as mean ± SD, *n* = 3. *P < 0.05 vs control. B, Effect of P‐STAT3 inhibitor treatment. C4‐2 and 22Rv1 cells were pretreated with S3i‐201(P‐STAT3 inhibitor) for 24 hours and subsequently coculture with CD4+ T cells for an additional 24 hours, and then treated with 3 nM Docetaxel for 48 hours. Finally, the apoptosis‐related protein levels were detected by Western blot analysis. The P‐STAT3 inhibitor treatment can reverse CD4+ T‐cell‐induced chemoresistance of PCa. Data are also presented as mean ± SD, *n* = 3. *P < 0.05 vs control. And ns is no statistical difference. CCL5, C‐C motif chemokine ligand 5; GAPDH, glyceraldehyde 3‐phosphate dehydrogenase; PCa, prostate cancer; STAT3, signal transducer and activator of transcription 3

**Figure 6:**
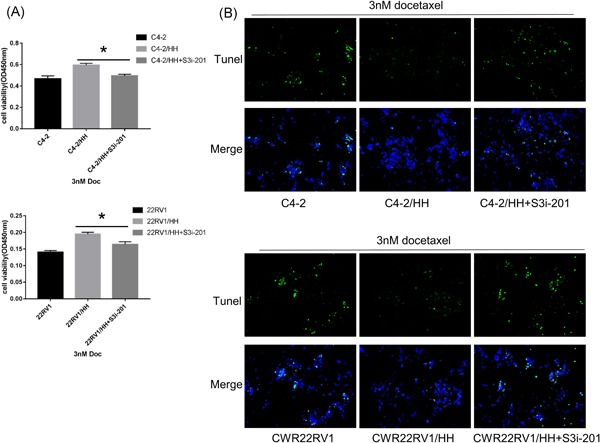
CD4+ T cells induced chemoresistance of PCa via CCL5/P‐STAT3 signaling. A, B, Effect of P‐STAT3 inhibitor treatment. Cell viability was detected using CCK8, Data are presented as mean ± SD, *n* = 3. *P < 0.05 vs control. and cell apoptosis was detected by Tunel assay. C4‐2 and 22Rv1 cells were pretreated with S3i‐201 (P‐STAT3 inhibitor) for 24 hours and subsequently coculture with CD4+ T cells for an additional 24 hours, and then treated with 3 nM Docetaxel for 48 hours. The P‐STAT3 inhibitor treatment can reverse CD4+ T cells induced chemoresistance of PCa from CCK8 and Tunel assay results. CCK8, cell counting kit‐8; CCL5, C‐C motif chemokine ligand 5; PCa, prostate cancer; STAT3, signal transducer and activator of transcription 3; Tunel, terminal deoxynucleotidyl transferase dUTP nick‐end labeling [Color figure can be viewed at wileyonlinelibrary.com]

To dissect the molecular mechanisms of how CD4+ T cells alter chemosensitivity of PCa, we focused on the STAT3 signals, which have demonstrated that constitutively activated phosphorylation of STAT3 (P‐STAT3) contributed to the development of tumor growth, drug resistance, and angiogenesis.^17^, ^18^ In addition, the P‐STAT3 signaling pathway can be activated via chemokines or cytokines, such as CCL5.^17^, ^18^, ^19^, ^20^, ^21^ P‐STAT3 was increased in PCa cells after coculture with CD4+ T cells, the similar results were obtained in C4‐2 and CWR22RV1 cells with CCL5 treatment (Figure 5A). Moreover, preincubation of C4‐2/CWR22Rv1 cells with 10 μM S3i‐201, a STAT3 inhibitor, could abolish the CD4+ T cells capacity to increase PCa chemoresistance by Western blot analysis (Figure 5B), Tunel (Figure 6A), and CCK8 (Figure 6B).

### 3.4 Infiltrated CD4 + T cells promote PCa chemoresistance in an in vivo mouse model

**Figure 7:**
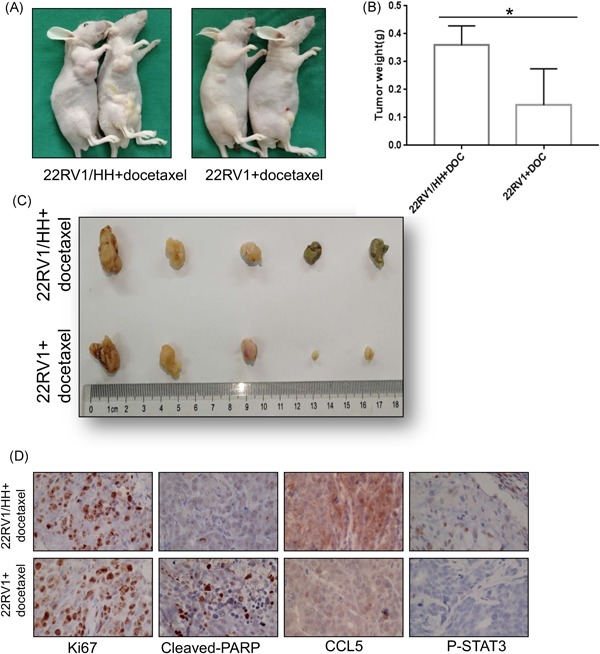
CD4+ T cells enhance PCa cell chemotherapy resistance in an in vivo mouse study. Ten male nude mice were injected subcutaneously with 2 × 10^6^ CWR22RV1 cells or 2 × 10^6^ CWR22RV1 cocultured with 2 × 10^5^ HH cells. After 2 weeks, the mice were treated with Docetaxel for three weeks and then killed. A, Gross appearance of the tumor xenografts. B, C, Weights of the xenografts tumor and macroscopic appearance of the xenografts tumor. Data are presented as mean ± SD, *n* = 5, *P < 0.05 vs control. D, IHC staining for Ki67, CCL5, P‐STAT3, and cleaved PARP in mice tumor tissues. CCL5, C‐C motif chemokine ligand 5; IHC, immunohistochemistry; PCa, prostate cancer; PARP, poly ADP ribose polymerase; STAT3, signal transducer and activator of transcription 3 [Color figure can be viewed at wileyonlinelibrary.com]

To confirm the above in vitro results from various cell lines studies, we then investigated the effects of infiltrated CD4+ T cells on PCa chemosensitivity using in vivo mouse PCa xenografted models with PCa CWR22RV1 cells coimplanted with T cells (HH) at 10:1 ratio. The tumor weight was assessed after Doc treatment for 4 weeks. After Doc administration, CWR22Rv1 tumor volumes were two‐fold larger in mice bearing CWR22Rv1 and HH cells than those in mice bearing CWR22Rv1 tumors alone (Figure 7A‐C).

To assess the role of CD4+ T cell‐induced chemoresistance of PCa tissues, we detected the expression of molecules examined in vitro through IHC, such as Ki67, P‐STAT3, cleaved PARP, and CCL5 in the xenograft tumors, and results were consistent with the findings in vitro (Figure 7D). Moreover, similar results were obtained when we replace CWR22RV1 cells with C4‐2 cells in vivo mouse study (Figure S2).

## 4 DISCUSSION

Doc‐based treatment is the first‐line chemotherapy for metastatic CRPC since 2004; however, chemoresistance remains a big challenge for the current PCa therapy.^1^, ^2^, ^3^, ^4^ Especially the mechanism of resistance to Doc is poorly understood. In the current study, firstly, we recruited patients showed significantly PSA increase, multiple or new metastases after Doc treatment, which were considered to be resistant to chemotherapy. In this part of the clinical specimens, CD4+ T‐cell infiltration in the tumor area was significantly increased. Then we further studied the existence of the relationship between CD4+ T‐cell infiltration and Doc resistance in PCa.

Interactions between cancer cells and the surrounding inflammatory microenvironment as well as the secreted cytokines and growth factors, such as IL8 and CCL2, play key roles in the development of Doc resistance.^22^, ^23^, ^24^, ^25^ Moreover, anti‐inflammatory agent resulted in more enhanced sensitivity of PCa cells to Doc‐induced apoptosis and improve the efficacy of Doc.^26^, ^27^ In the tumor inflammatory microenvironment, CD4+ T cells are important components and play an important role in PCa progression. McArdle et al^28^ showed that the presence of increased CD4+ T‐lymphocyte infiltrate was associated with poor PCa prognosis, and it can enable survival prediction. Wenner et al^29^ reported that Doc induced an increase in both CD4+ and CD8+ TILs over saline treatment in transgenic adenocarcinoma of the mouse prostate‐C2‐bearing mice. But the role of CD4+ T cells in chemoresistance in PCa still remains unclear. Here, we demonstrated the consequence of infiltrating CD4+ T cells could enhance PCa cell Doc resistance in vitro study and in the mouse model.

We also found the key cytokine—CCL5, also known as RANTES which is the most secreted factor by CD4+ T cells after cocultured with PCa cells. It is one of the members of the CC chemokine family of proteins, and upregulation of CCL5 can increase the aggressive potential of PCa cells and the size of PCa stem cell populations.^30^, ^31^, ^32^, ^33^ Moreover, it has also been verified that overexpression of CCL5 facilitates tumor progression^19^, ^21^ and can activate PI3K/Akt and STAT3 signaling pathway, which plays a vital role in tumor progression, adhesion, and drug resistance in breast and ovarian cancers.^17^, ^18^, ^20^ CCL5, as previously shown,^32^ directly increased PCa cell migration.

To further study the mechanism of CD4+ T cell and the secreted CCL5 promoting PCa cells chemotherapy‐resistant, we then focused on the STAT3 signaling pathway, a member of the STAT transcription factor family. In PCa, STAT3 activation correlates with Gleason score and pathological stage,^34^, ^35^ promotes tumor invasion and metastasis,^36^ is involved in resistance to enzalutamide^37^ and Doc.^38^ Here, we found that CD4+ T cells might affect PCa chemosensitivity through CCL5 activation of STAT3 via upregulation of STAT3 phosphorylation.

## 5 CONCLUSIONS

**Figure 8:**
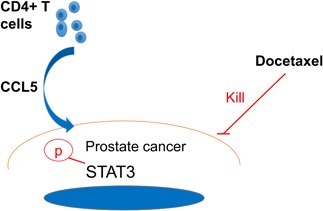
Mechanisms of CD4+ T cells to promote PCa cell chemoresistance. CCL5, C‐C motif chemokine ligand 5; PCa, prostate cancer; STAT3, signal transducer and activator of transcription 3 [Color figure can be viewed at wileyonlinelibrary.com]

The most striking finding of the present study was that infiltrating CD4+ T cells could promote PCa chemotherapy resistance via modulation of the CCL5/STAT3 signaling pathway (Figure 8). Therefore, STAT3 knockdown, administration of certain small molecule inhibitors, or CCL5 blockade by using neutralizing antibody in combination with the conventional Doc treatment may provide a new therapeutic approach for PCa patients with acquired Doc resistance.

## CONFLICT OF INTERESTS

The authors declare that there are no conflict of interests.

## Supporting information

Supporting information

Supporting information

Supporting information
