# Supplementary figures and images for: Infiltrating CD4+ T cells attenuate chemotherapy sensitivity in prostate cancer via CCL5 signaling
Source: Prostate. 2019 Apr 24;79(9):1018–31. doi: 10.1002/pros.23810 (PMC6594129; doi:10.1002/pros.23810)

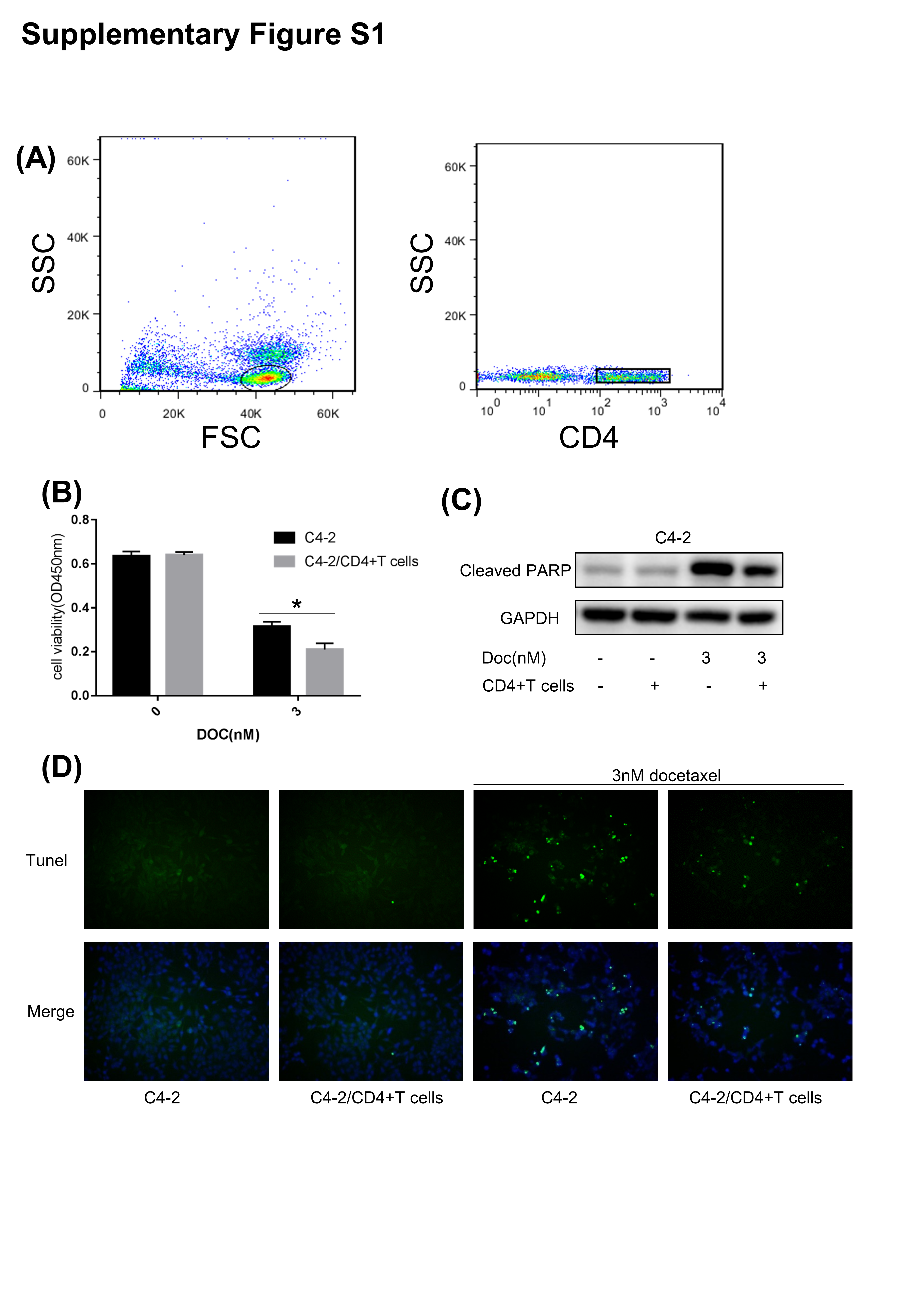

Supplement: Supplementary file 1 — Supporting information [file PROS-79-1018-s001.tif]

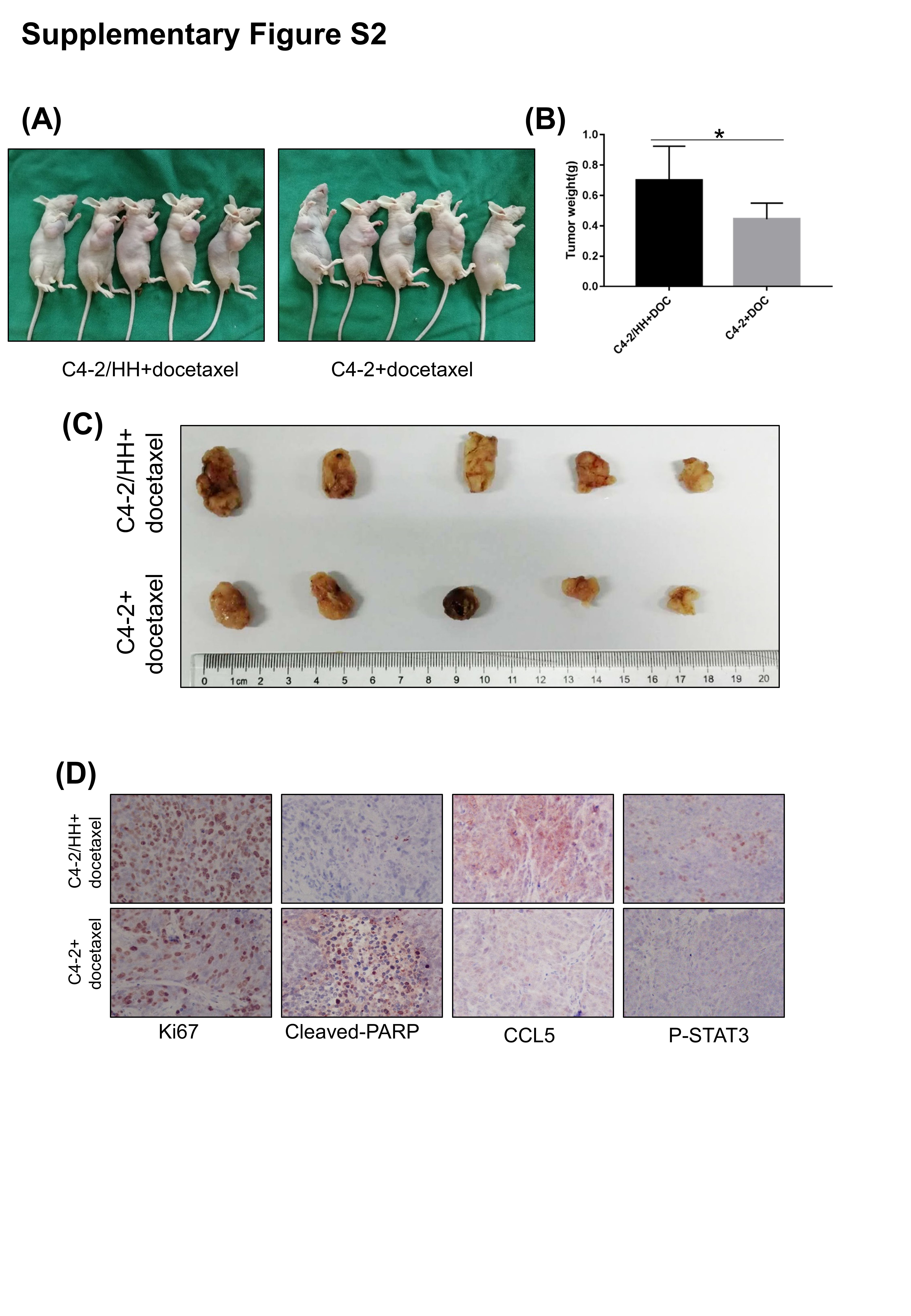

Supplement: Supplementary file 2 — Supporting information [file PROS-79-1018-s002.tif]
