# Supplementary material for: Infiltrating CD4+ T cells attenuate chemotherapy sensitivity in prostate cancer via CCL5 signaling
Source: Prostate. 2019 Apr 24;79(9):1018–31. doi: 10.1002/pros.23810 (PMC6594129; doi:10.1002/pros.23810)
Supplement: Supplementary file 3 — Supporting information [file PROS-79-1018-s003.Docx]

**Supplementary Figure S1. CD4+T cells altered PCa chemotherapy sensitivity**

**A.** Isolation of CD4+T cells from peripheral blood mononuclear cells(PBMCs) using flow cytometry. **B.** C4-2 cells were co-cultured with CD4+T cells for 3 days in 24-well plates under 0.4 μm transwell membranes. Subsequently, C4-2 cells were treated with 3nM Doc (docetaxel) for 48 h and tested with CCK8. Data are presented as mean± SD, n=3, **P<0.05* versus control. **C.** Western blot analysis: for docetaxel treatment, C4-2 cells co-cultured with CD4+T cells showed less expression of cleaved-PARP compared to C4-2 cells without co-culture CD4+T cells. **D.** TUNEL assay analysis of cell apoptosis. For 3nM docetaxel treatment, C4-2 cells co-cultured with CD4+T cells showed less cell apoptosis compared to C4-2 cells without co-culture CD4+T cells.

**Supplementary Figure S2. CD4+T cells enhance PCa cell chemotherapy resistance in an in vivo mouse study**.

Ten male nude mice were injected subcutaneously with 2×10^6^ C4-2 cells or 2×10^6^ C4-2 co-cultured with 2×10^5^ HH cells. After about one week, the mice were treated with docetaxel every five days for three treatments and then sacrificed. **A.** Grossly appearance of the tumor xenografts. **B-C.** Weights of the xenografts tumor and macroscopic appearance of the xenografts tumor. Data are presented as mean± SD, n=5 **P<0.05* versus control. **D.** IHC staining for Ki67, CCL5, P-STAT3, and cleaved-PARP in mice tumor tissues.
